# Mechanisms of photobiomodulation therapy in treating and preventing antineoplastic-induced oral mucositis: a systematic review

**DOI:** 10.1590/acb403125

**Published:** 2025-03-31

**Authors:** Paulo Victor Alves de Sales, Isabella Piassi Dias Godói, Gerly Anne de Castro Brito, Renata Carvalho Leitão, Aurigena Antunes de Araújo, Caroline Addison Carvalho Xavier de Medeiros

**Affiliations:** 1 Universidade Federal do Rio de Janeiro – Institute of Pharmaceutical Sciences – Multidisciplinary Center of Macaé – Macaé (RJ) – Brazil.; 2 Universidade Federal do Rio de Janeiro – Health Technology Assessment Center – Management, Economics, Health Education and Pharmaceutical Services – Rio de Janeiro (RJ) – Brazil.; 3 Universidade Federal do Ceará – Faculty of Medicine – Post Graduate Program of Morphofunctional Sciences – Fortaleza (CE) – Brazil.; 4 Universidade Federal do Rio Grande do Norte – Department of Biophysics and Pharmacology – Post Graduate Program in Pharmaceutical Science – Natal (RN) – Brazil.; 5 Universidade Federal do Rio Grande do Norte – Department of Biophysics and Pharmacology – Post Graduate Program Dental Sciences – Natal (RN) – Brazil.; 6 Universidade Federal do Rio Grande do Norte – Post Graduate Program Biotechnology, Northeast Biotechnology Network – Natal (RN) – Brazil.; 7 Universidade Federal do Rio Grande do Norte – Post Graduate Program Biochemistry and Molecular Biology – Natal (RN) – Brazil.

**Keywords:** Stomatitis, Low-Level Light Therapy, Drug Therapy, Radiotherapy

## Abstract

**Purpose:**

To conduct a systematic review of the mechanisms of photobiomodulation therapy (PBMT) for treating or preventing oral mucositis (OM) caused by antineoplastic therapy.

**Methods:**

Following PRISMA 2020 guidelines, a search was conducted in Medline, Latin American and Caribbean Health Sciences Literature (LILACS), Scientific Electronic Library Online (SciELO), and Bibliografia Brasileira de Odontologia from August to September 2023 using descriptors related to OM and laser therapy. Studies on the mechanisms of photobiomodulation in OM were included. Randomized (RCTs) or non-randomized trials from the past 10 years were reviewed. Risk of bias was assessed using RoB 2.0 and ROBINS-I tools.

**Results:**

A total of 355 studies was identified. After the screening, seven met the eligibility criteria. The RCTs showed a low risk of bias. PBMT reduced OM incidence in patients undergoing chemotherapy/radiotherapy. PBMT decreased pro-inflammatory cytokines (interleukin-6, tumor necrosis factor-α) and increased anti-inflammatory cytokines (interleukin-4, interleukin-10). It also modulated inflammatory mediators, enhancing the antioxidant enzyme superoxide dismutase and overexpressing genes for keratinocyte differentiation, aiding injury repair.

**Conclusion:**

The findings suggested that the mechanism of action of PBMT in OM involves modulation of the inflammatory response, balancing oxygen reactive species generation, and expression of factors related to healing or repair. Further studies are needed to elucidate these mechanisms and optimize treatment protocols.

## Introduction

Oral mucositis (OM) is a lesion in the oral mucosal. Initially, it is characterized as erythema, progressing to atrophy and ulceration, often with pseudomembranous formations^1^. This condition affects patients undergoing cancer chemotherapy (CT) and/or head and neck radiotherapy (RT) in cancer treatmen^t2,^ 3. In a clinical context, the incidence of OM in patients undergoing chemotherapy is 40%^4^. In protocols that combine CT and RT, the incidence of mucositis rises to 90–100%^5^. The OM can lead to complications for the patient, such as severe pain, difficulty eating, speaking, and swallowing, and an increased risk of infections6. Thus, OM harms patients’ quality of life and can compromise the efficacy of antineoplastic treatment due to interruptions or reductions in the doses of CT or RT^6^^,^ 7.

The OM pathophysiology is a complex and dynamic process involving five phases: initiation, damage response, amplification, ulceration, and healing. Mucositis begins with damage caused by antineoplastic therapy (CT and/or RT), leading to DNA breaks and a local increase in reactive oxygen species (ROS)^6^, resulting in the activation of the nuclear transcription factor factor nuclear kappa B (NF-kB)^6^^,^ 8 ^–^ 10. NF-kB regulates over 200 genes, including critical pro-inflammatory cytokines such as tumor necrosis factor (TNF)-α, interleukin (IL)-1β, and IL-6. The release of these pro-inflammatory mediators results in a positive feedback loop that continuously activates NF-kB, causing signal amplification and prolonging the damage to the oral mucosa, ultimately leading to epithelial ulceration^6^^,^ 8 ^–^ 10.

The ulcerative phase is the most symptomatic OM stage. It presents an increased risk of secondary infections, as the ulcers serve as focal points for colonization by microorganisms^6^^,^ 8. Healing typically begins about two weeks after the conclusion of antineoplastic therapy, and this phase is characterized by the proliferation of the epithelium around the ulcers and the restoration of the local microbiota. Additionally, symptoms of oral mucositis may persist for as long as eight weeks following the end of treatment^11^.

The treatment of OM in oncology patients aims to relieve symptoms, prevent or treat infections, maintain adequate hydration and nutrition, and avoid interruptions or reductions in CT or RT doses^7^. Among the available therapeutic modalities, photobiomodulation therapy (PBMT) stands out as a widely adopted intervention for managing OM, being recommended in clinical practice guidelines for the management of mucositis secondary to cancer therapy by the Multinational Association of Supportive Care in Cancer and the International Society of Oral Oncology. PBMT stimulates cells with a sufficiently low-energy density to avoid thermal damage to tissues, triggering anti-inflammatory, analgesic, and modulatory effects^12^^–^ 15.

It is suggested that the effects of laser therapy are predominantly mediated by photoactivation cellular mechanisms, primarily through the absorption of light by mitochondrial cytochrome c oxidase^12^^,^ 15 ^,^ 16. This absorption triggers cellular signaling that stimulates protein synthesis and cell proliferation^12^^,^ 15 ^,^ 16. It is important to note that the underlying mechanisms of the therapeutic effects of laser therapy on analgesia and tissue repair are not fully understood yet, although recent studies have corroborated its efficacy in treating various clinical conditions^17^^,^ 18. Therefore, the present study aimed to conduct a systematic review to identify photobiomodulation’s mechanisms or signaling pathways in treating or preventing OM in oncology patients.

## Methods

### Study design

The study protocol was registered and accepted in the International Prospective Register of Systematic Reviews (PROSPERO), under CRD42023468625. Additionally, this systematic review complies with the PRISMA 2020 statement^19^. This study was carried out at the Institute of Pharmaceutical Sciences, Multidisciplinary Center of Macaé, Universidade Federal do Rio de Janeiro, Macaé, RJ, Brazil.

### Search strategy

A search was conducted in the Medline, Latin American and Caribbean Health Sciences Literature (LILACS), Scientific Electronic Library Online (SciELO), and Bibliografia Brasileira de Odontologia databases using the following descriptors: “Oral Mucositis,” “Mucositis,” “Stomatitis,” “Photobiomodulation,” “Chemotherapy,” “Radiotherapy,” and “Chemoradiotherapy.” The keywords were combined using the terms “AND” and “OR” during the database searches.

### Study selection & eligibility criteria

The guiding question of this systematic review was: “What are the mechanisms of action of PBMT in preventing and/or treating OM in patients undergoing CT and/or RT?”^20^. Using the P(opulation) I(ntervention) C(ontrol/comparator) O(utcomes) acronyms, the population consisted of patients who underwent PBMT in clinical trials investigating the therapy’s mechanisms of action. The intervention evaluated was PBMT as an adjunctive treatment for OM during or after cancer therapy, compared to patients with OM who did not receive this treatment. The outcomes of interest also included the expression of genes, growth factors, proteins, or inflammatory mediators related to oral mucositis. Randomized clinical trials (RCTs) and non-randomized trials published in the last 10 years (2013–2023) were included as inclusion criteria.

Exclusion criteria included observational studies, case reports, *in-vitro* studies, animal studies, reviews, and case series. Studies that lacked full-text availability or provided insufficient data for analysis were also excluded. Two independent reviewers (P.S. and C.M.) evaluated the titles, abstracts, and full-text studies, with a third reviewer (I.G.) resolving any discrepancies.

### Data analysis

For the articles that met the inclusion criteria, a detailed review was conducted to collect information on various variables of interest: study design, type of laser used, laser power and wavelength, type of cancer, biomarkers examined, mechanism of action, and antineoplastic therapy. Two researchers independently extracted the data, and disagreements were resolved through discussions to ensure the information was accurate.

### Quality assessment of the studies: risk of bias

The methodological quality of the studies was evaluated using the RoB 2.0 tool for RCTs and the ROBINS-I tool for non-randomized trials. RoB 2.0^21^, visualized in the RevMan 5.4 software, assessed five domains: randomization process, deviations from intended interventions, missing outcome data, outcome measurement, and selective outcomes reporting. The ROBINS-I tool^22^ evaluated seven domains: confounding, participant selection, classification of interventions, deviations from intended interventions, missing data, outcome measurement, and selective outcomes reporting. Each domain was categorized as having a low, moderate, serious, or critical risk of bias.

## Results

### Selected studies

**Figure 1:**
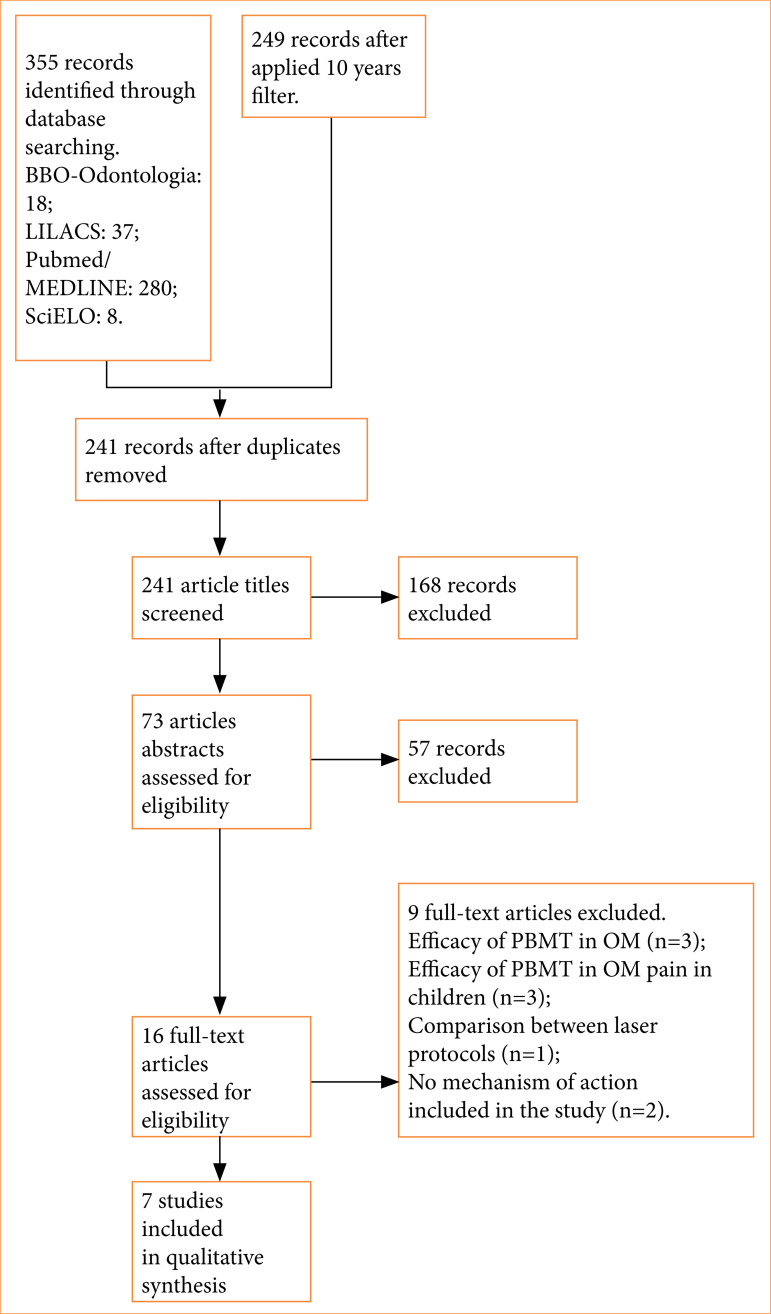
PRISMA flowchart.

LILACS: Latin American and Caribbean Health Sciences Literature; SciELO: Scientific Electronic Library Online; BBO: Bibliografia Brasileira de Odontologia; OM: oral mucositis; PBMT: photobiomodulation therapy. Source: Elaborated by the authors.

As shown in the PRISMA flowchart (Fig. 1), 355 articles were identified for the present study. After removing duplicates and screening titles and abstracts, 16 were selected for full-text review. Nine were excluded because they did not meet the pre-defined study eligibility criteria. Consequently, seven articles were selected for data extraction.

### Characterization of the studies

The main characteristics of the selected studies are listed in Table 1. The articles were published between 2014 and 2021. Among these, three were RCTs, one was a quasi-experimental clinical trial, one was a retrospective study, one was a preliminary RCT, and one was a pilot clinical trial. The RCTs studied inflammatory mediators, metalloproteinases, and growth factors involved in the effects of PBMT in OM therapy. The studies by Lessa^25^, Martins et al.^26^, and Oton-Leite et al.^27^ used an InGaAlP diode laser (Twin Flex Evolution, MMOptics LTDA, São Carlos, SP, Brazil) operating at 660 nm with the dose of 6.2 J/cm^2^. In the preliminary RCT by Antunes et al.^23^, an InGaAlP diode laser (Photon Lase III) with frequency of 660 nm and the dose of 4 J/cm^2^ was used.

**Table 1:** Characteristics of the selected studies.

Study (Year)	Study design	Intervention		Biomarkers	n	
		Laser	Power		Control	Intervention
Antunes et al. (2017)^23^	Preliminary randomized clinical trial	InGaAlP diode laser (660 nm)	4 J/cm^2^ (1,7 E)	Genes linked to cytotoxicity, immune response, and keratinocyte differentiation	14	13
Menezes et al. (2021)^24^	Retrospective study	AlGaAs diode laser (630 nm)	2 J/cm^2^ (0,9 E)	SOD	83	204
Lessa (2016)^25^	Randomized clinical trial	AlGaAs diode laser (660 nm)	A: 3,8 J/cm^2^ (1,6 E) B: 6,3 J/cm^2^ (2,6 E) C: 3,8 J/cm^2^ (1,6 E)	Genes linked to the immune response	7	Group A = 10 Group B = 9
Martins et al. (2020)^26^	Randomized clinical trial	InGaAlP diode laser (660 nm)	6,2 J/cm^2^ (2,6 E)	Nitrites, IL-6, IL-8, IL-10, IL-12p70, IL-1β and TNF-α	23	25
Oton‐Leite et al. (2015)^27^	Randomized clinical trial	InGaAlP diode laser (660 nm)	6,2 J/cm^2^ (2,6 E)	TNF-α, IL-6, IL-1β, IL-10, TGF-β, EGF, FGF, VEGF, MMP2/TIMP2 and MMP9/TIMP2	13	12
Rezk-Allah et al. (2019)^28^	Quasi-experimental study	GaAs diode laser (904 nm)	1 J/cm^2^ (0,3 E)	TNF-α and IL-6	-	80
Zanotta et al. (2020)^29^	Preliminary clinical trial	AlGaAs diode laser (907 nm)	6 J/cm^2^ (1,9 E)	38 cytokines and chemokines*	-	4

SOD: superoxide dismutase; IL: interleukin; TNF: tumor necrosis factor; TGF: tumor necrosis factor; EGF: epidermal growth factor; FGF: fibroblast growth factor; VEGF: vascular endothelial growth factor; MMP2/TIMP2: matrix metalloproteinase 2 / tissue inhibitor of metalloproteinases 2; *IL-1β, IL-2, IL-4, IL-5, IL-6, IL-7, IL-8, IL-10, IL12(p70), IL-13, IL-17, granulocyte colony-stimulating factor (G-CSF), interferon gamma (IFN-γ), monocyte chemoattractant protein-1 (MCP-1), macrophage inflammatory protein-1 beta (MIP-1β), RANTES (Activation-Regulated, Normal T Expressed and Secreted), TNF-β, IL-1α, interleukin receptor alpha-2 (IL-2Rα), IL-3, IL-12(p40), IL-16, IL-18, cutaneous T-cell attractive chemokine (CTACK), growth-regulated oncogene alpha (GRO-α), hepatocyte growth factor (HGF), interferon alfa-2 (IFN-α2), leukemia inhibitory factor (LIF), monocyte chemoattractant protein-3 (MCP-3), macrophage colony-stimulating factor (M-CSF), macrophage migration inhibitory factor (MIF), interferon gamma induced chemokine (MIG), nerve growth factor beta (βNGF), stem cell factor (SCF), stem cell growth factor-beta (SCGF-β), stromal cell-derived factor 1 alpha (SDF-1α), TNF-α, and TNF-related apoptosis inducing ligand (TRAIL); SOD - superoxide dismutase. E - Einstein unit refers to the energy applied to the surface of biological tissue^45^. Source: Elaborated by the authors.

The non-randomized clinical trials investigated the effects of PBMT in OM therapy, focusing on inflammation mediators, cytokines, and the activity of antioxidant enzymes. Rezk-Allah et al.^28^ evaluated serum levels of TNF-α and IL-6 in response to PBMT treatment using a GaAs diode laser (904 nm, 1 J/cm^2^). Menezes et al.^24^ analyzed superoxide dismutase (SOD) activity and leukocyte count following treatment with a diode laser (630 nm, 2 J/cm^2^). Zanotta et al.^29^ investigated the levels of 38 cytokines and chemokines in oral samples collected before, and after the final session, and the day after the treatment with an AlGaAs diode laser (907 nm, 6 J/cm^2^).

### Mechanisms of action

Regarding the mechanisms of action or signaling pathways, most studies demonstrated changes in the expression of pro- and anti-inflammatory cytokines (Table 2). Additionally, the antioxidant effect and the expression of factors related to tissue healing or repair were investigated as potential molecular mechanisms underlying the action of PBMT. These findings suggested that PBMT modulates the inflammatory response and enhances tissue regeneration, a crucial process in mitigating the effects of CT and RT-induced mucositis.

**Table 2:** Proposed mechanisms for the action of photobiomodulation therapy in oral mucositis.

Mechanism	Studies
Modulation of inflammatory response	Antunes et al.^23^, Lessa^25^, Martins et al.^26^, Oton‐Leite et al.^27^, Rezk-Allah et al.^28^, Zanotta et al.^29^
Antioxidant effect	Menezes et al.^24^
Tissue repair	Antunes et al.^23^

Source: Elaborated by the authors.

### PBMT in modulating the inflammatory response

Rezk-Allah et al.^28^ reported a significant decrease in the average grade of mucositis, from 2.35 ± 0.695 to 1.13 ± 0.333, following PBMT treatment (*p* < 0.001). The clinical trial also identified a significant reduction in serum TNF-α levels in breast cancer patients (pre-PBMT = 82.13 ± 14.54 pg/mL; post-PBMT = 17.05 ± 2.267 pg/mL; *p* = 0.0045). However, this effect was not observed in patients with head and neck cancer or non-Hodgkin lymphoma. Additionally, there was a reduction in serum IL-6 levels in both head and neck cancer patients (pre-PBMT = 94.10 ± 27.17 pg/mL; post-PBMT = 17.30 ± 2.676 pg/mL; *p* = 0.0307) and breast cancer patients (pre-PBMT = 99.48 ± 21.74 pg/mL; post-PBMT = 26.93 ± 6.813 pg/mL; *p* = 0.0190) (Table 3). Furthermore, Oton-Leite et al.^27^ observed a significant reduction in salivary IL-6 concentration by the end of RT (35th session) in patients undergoing PBMT compared to the control group. In addition, fibroblast growth factor levels significantly decreased following the ulcerative phase of OM in the laser-treated group, corresponding to the healing process.

**Table 3:** Proposed mechanisms for the action of photobiomodulation therapy in the modulation of the inflammatory response.

Study (year)	Effects	Mechanisms
Antunes et al. (2017)^23^	↑ SPRR2B and SPRR2F ↓ HLA-A, HLAC and HLA-DP	Increased direct or indirect expression of genes involved in keratinocyte differentiation and inhibition of pro-inflammatory cytokine release.
Lessa (2016)^25^	↑ IL-10, IL-1A and LY96 ↓ IL-6 and MIF	Increased direct or indirect expression of genes involved in immune response modulation, promoting an improvement in the inflammatory condition.
Martins et al. (2020)^26^	↑ IL-10, IL12p70 and TNF-α	Balance in the inflammatory response and stimulation of antigen-presenting cells expressing TLRs, promoting a more effective immune response against microorganisms.
Oton‐Leite et al. (2015)^27^	↓ IL-6 and FGF	-
Rezk-Allah et al. (2019)^28^	↓ IL-6 and TNF-α	-
Zanotta et al., (2020)^29^	↑ IL-4 and IL-10	Immunomodulatory effect, promoting balance in the inflammatory response.

IL: interleukin; FGF: fibroblast growth factor; LY96: lymphocyte antigen 96; MIF: migration inhibitory factor; TNF: tumor necrosis factor; HLA-A: human leukocyte antigen – A; HLAC: human leukocyte antigen – C; HLA-DP: human leukocyte antigen – DP; TLR: toll-like receptor. Source: Elaborated by the authors.

On the other hand, Martins et al.^26^ identified higher salivary concentrations of the pro-inflammatory cytokines IL-12p70 and TNF-α in the laser-treated group (1.20 pg/mg and 0.43 pg/mg, respectively) compared to the control group (0.0 pg/mg). Additionally, the authors reported increased concentrations of the anti-inflammatory cytokine IL-10 in the PBMT group (2.51 pg/mg) compared to the control group (0.86 pg/mg) at the end of the laser treatment.

Zanotta et al.^29^ identified a significant increase in the pro-inflammatory cytokines IFN-α2, IL-18, and migration inhibitory factor (MIF) in response to PBMT in ulcerated areas during stages T4 and T5. This trend was observed in patients ID 1-3, who showed a positive clinical response to laser therapy. In contrast, patient ID 4, who did not clinically respond to the treatment, did not exhibit the same increase in these cytokines. Additionally, global anti-inflammatory cytokines such as IL-4, IL-10, IL-13, granulocyte colony-stimulating factor, leukemia inhibitory factor, and stem cell growth factor-β were increased in the ulcerated areas of patients ID 1-3 in response to PBMT.

Lessa^25^ investigated the effects of PBMT at the molecular level using a different approach by analyzing gene expression in the various experimental groups. In group A (15 mW/3.8 J/cm^2^), there was a significant increase in the expression of the IL-10 gene. Conversely, a reduction was observed in the CD4, CD86, IL-6, MIF, NFATC3, PTPRC, and SMAD3 gene expression, suggesting a suppression of genes associated with the inflammatory and immune response. In group B (25 mW/6.3 J/cm^2^), genes such as BAX, CCL2, FAS, HMOX1, ICAM1, IL-1A, NFATC3, and TGFB1 were increased expressed in response to higher-intensity laser treatment, indicating a response to abiotic and oxidative stress. The genes CD80, IKBKB, IL-6, and SKI were silenced. Regardless the laser power, the IL-1A and lymphocyte antigen 96 (LY96) gene expression were increased compared to the control group (group C). Moreover, both groups A and B demonstrated increased expression of genes related to the response to bacterial lipopolysaccharides (CD68, ICAM1, LY96, STAT3, and TGFB1), indicating a potential enhancement in the inflammatory response and improved cellular resistance to pathogens.

### Antioxidant activity of PBMT

The antioxidant mechanisms of PBMT are presented in Table 4. When comparing SOD levels before the first cycle and after the last cycle of CT, Menezes et al.^24^ observed a significant reduction in SOD activity in patients who were not subjected to PBMT (pre-CT = 1.89 ± 0.66 U/mL; post-CT = 0.691 ± 0.53 U/mL; *p* < 0.05). In contrast, the reduction in SOD activity was prevented in the PBMT-treated group (pre-CT = 1.652 ± 0.77 U/mL; post-CT = 1.404 ± 0.66 U/mL; *p* < 0.05). Furthermore, the incidence of OM was lower (14.7%) in the PBMT group compared to the control group (69.9%), with reduction in incidence observed across all chemotherapy protocols analyzed in the study.

**Table 4:** Proposed mechanisms for the antioxidant action of photobiomodulation therapy.

Study (Year)	Effects	Mechanisms
Menezes et al. (2021)^24^	↑ SOD	Antioxidant effect through the preservation of SOD activity.

SOD: superoxide dismutase. Source: Elaborated by the authors.

### PBMT and tissue repair

The DNA microarray analysis by Antunes et al.^23^ identified 165 expressed genes in the samples from patients treated with PBMT. Among them, 105 genes were upregulated, and 60 genes were downregulated. Between the upregulated genes, SPRR2B had the highest fold change (13×), followed by PPIAL4B (4.6×), and LCE3D (4.0×). When it comes to downregulated genes, the three with the most significant fold change were TMPRSS11E (3.8×), FAM60A (3.1×), and SNORD116-6 (2.7×). The authors reported that the upregulated genes are involved in biological pathways related to keratinocyte differentiation, epidermal cell differentiation, skin development, and epidermis development. In contrast, the downregulated genes are associated with the positive regulation of T-cell-mediated cytotoxicity (Table 5).

**Table 5:** Proposed mechanisms for the action of photobiomodulation therapy in tissue repair.

Study (Year)	Effects	Mechanisms
Antunes et al. (2017)23	↑ SPRR2B e SPRR2F ↓ HLA-A, HLAC and HLA-DP	Increased expression, directly or indirectly, of genes involved in keratinocyte differentiation and inhibition of the release of pro-inflammatory cytokines.

HLA-A: human leukocyte antigen – A; HLAC: human leukocyte antigen – C; HLA-DP: human leukocyte antigen – DP. Source: Elaborated by the authors.

The real-time quantitative polymerase-chain-reaction (qPCR) analysis identified that the HLA genes (HLA-A, HLA-C, and HLA-DP), associated with leukocyte-mediated cytotoxicity and T-cell-mediated immunity, were overexpressed in the placebo group. Moreover, after validation with qPCR, the SPRR2B and SPRR2F genes were classified as differentially expressed in patients undergoing PBMT. Compared to the placebo group, the study demonstrated that patients treated with PBMT exhibited changes in gene pathways related to keratinization and epidermal cell differentiation.

### Quality assessment and risk of bias

**Figure 2:**
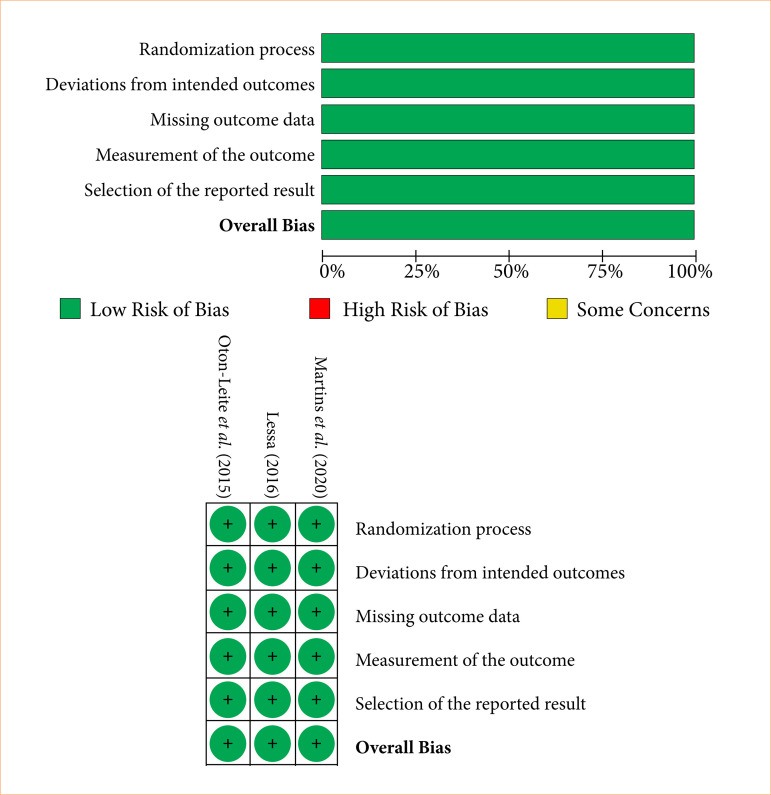
Risk of bias graph of randomized clinical trials (RoB 2.0).

Source: Elaborated by the authors.

The RCTs exhibited a low risk of bias in critical areas, including randomization and outcome measurement. The losses were reported to be balanced between the groups (Fig. 2). However, the preliminary study by Antunes et al.^23^ raised concerns about missing data and unclear details regarding randomization and allocation concealment, resulting in an uncertain risk of bias.

**Figure 3:**
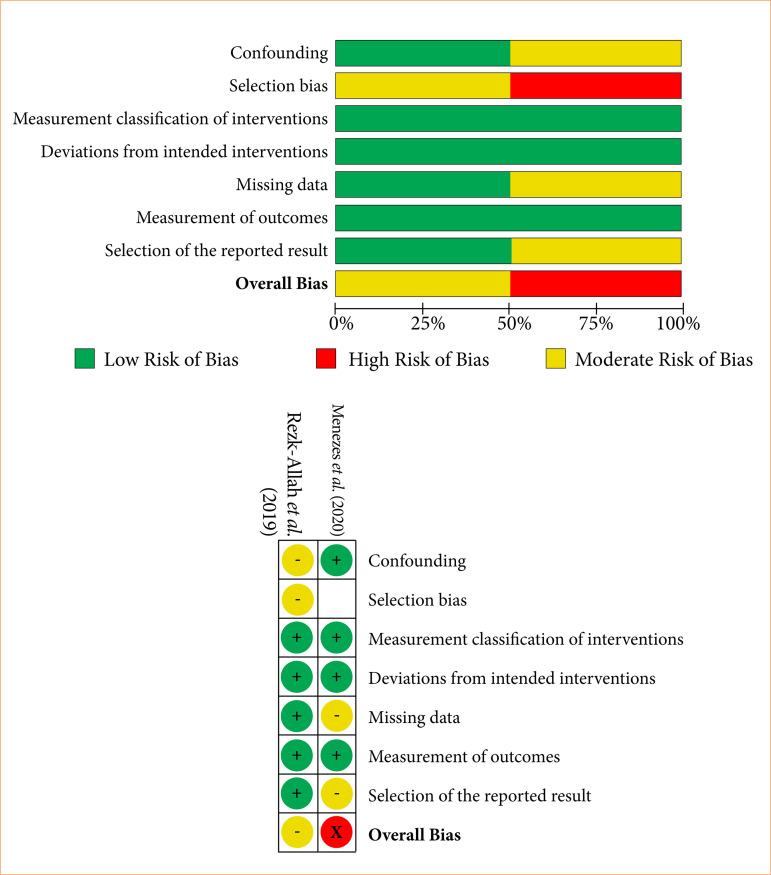
Risk of bias graph of non-randomized clinical trials (ROBINS-I).

Source: Elaborated by the authors.

Non-randomized trials presented moderate to serious risk of bias, primarily due to inconsistencies in confounding factors, participant selection, and missing data (Fig. 3). The study by Menezes et al.^24^ had a serious risk of selection bias due to the mismatch between collected samples and participants. Rezk-Allah et al.^28^ faced a moderate risk of confounding bias due to the inclusion of tumors at varying stages, while the pilot study by Zanotta et al.^29^ had a moderate risk of missing data but a low risk in other domains.

This assessment highlights the importance of addressing methodological weaknesses, especially in non-randomized studies, to ensure the reliability of results.

## Discussion

This study analyzed the current evidence on the mechanisms involved in the effects of PBMT in treating and preventing OM. OM is a complex and dynamic adverse reaction to cancer treatment, influenced by various factors. In this context, PBMT has been shown to modulate the inflammatory response, promote tissue repair, and reduce oxidative stress levels in the affected tissue^23^^–^ 29.

In the present study, the laser devices used in the articles are in accordance with the guidelines for the treatment of prevention of OM. The recommendation is a visible wavelength (630–680 nm) LED/laser device with power density (irradiance of the treatment surface) of 10–50 mW/cm2 for a total dose of 1.2 Einstein (photon fluence at 650 nm = 5.7 p·J/cm^2^) per treatment field carried out in 30 to 120 minutes before oncotherapy. Other wavelengths (400–1,100 nm) can be used. Furthermore, for OM treatments with a laser device, a total dose of 2.5 Einstein (photon fluency at 650 nm = 11.4 p·J/cm^2^) was repeated three or four times a week for at least 15–20 sessions or until healing after the end of oncotherapy^30^.

The pathophysiology of OM is complex and involves five stages: initiation, response to primary damage, signal amplification, ulceration, and healing, and several mediators are involved.^31^. Cytokines play a crucial role in the pathophysiology of OM induced by antineoplastic therapy^2^^,^ 3. Key pro-inflammatory cytokines, such as IL-6, IL-1β, and TNF-α, are involved in the onset and progression of OM, while anti-inflammatory cytokines like IL-4 and IL-10 are highlighted as protective factors against mucositis^23^^,^ 26 ^–^ 29. The reduction of IL-6 following PBMT treatment has been previously demonstrated in various studies, both *in-vitro* models of OM and in patients with periodontitis^32^^,^ 33. In the clinical trials, IL-6 showed a significant reduction in both serum and salivary levels by the end of PBMT treatment, indicating that laser therapy effectively modulates its expression during OM treatment^25^^,^ 27 ^,^ 28.

TNF-alpha is crucial in OM pathophysiology^1^^,^ 2. In the clinical trial conducted by Rezk-Allah et al.^28^, a reduction in serum TNF-alpha levels was observed in breast cancer patients treated with PBMT, aligning with previous findings from *in-vitro* and *in-vivo* experimental studies^34^^–^ 36. One notable contrast to this result is the increase in TNF-alpha after PBMT treatment observed by Martins et al.^26^. This discrepancy could be attributed to differences in laser therapy application, highlighting the importance of clinical context and treatment specificity in modulating inflammatory biomarkers. Rezk-Allah et al.’s study^28^ focused on using curative laser therapy (dose of 0.3 Einstein, photon fluence at 904 nm = 1 J/cm^2^) for individuals already experiencing OM, while Martins et al.’s study^26^ applied preventive laser therapy (dose of 2,6 Einstein, photon fluence at 660 nm = 6.2 J/cm^2^) to patients before OM had developed. Furthermore, the two studies used different methods for cytokine quantification, which may also explain the differences in their results.

Bacterial biofilm formation is a crucial factor in the progression of OM^2^^,^ 3. Martins et al.^26^ highlighted the positive modulation of IL-12p70, a cytokine closely associated with cytotoxic immune responses and antigen presentation^37^. This finding underscores the complexity of the immune response involved in OM and suggests that PBMT may directly influence underlying immunological mechanisms^26^.

Furthermore, the clinical research led by Lessa^25^ revealed that patients undergoing PBMT, regardless the protocol used, exhibited increased expression of LY96, an essential gene encoding a protein associated with toll-like receptor 4 (TLR4) on the cell surface. This protein is critical in responding to lipopolysaccharides (LPS), a component of bacterial biofilms^37^^,^ 38. These findings suggest that PBMT may significantly regulate the oral environment by modulating the immune response and influencing the host-biofilm interaction^25^^,^ 26.

Lessa^25^ and Martins et al.^26^ observed increased anti-inflammatory cytokines, such as IL-4 and IL-10, following PBMT treatment. This suggests a potential for balancing the immune and inflammatory responses, highlighting the clinical relevance of PBMT as a prophylactic approach for managing OM. These results offer new perspectives for treating and preventing this debilitating condition.

The relationship between oxidative stress and OM is well established, with oxidative stress playing a vital role in the pathogenesis of this conditio^n2,^ 8. In this context, the therapeutic effects of PBMT are being explored for their potential antioxidant mechanisms. In the clinical trial conducted by Menezes et al.^24^, SOD activity was evaluated in patients undergoing PBMT for the prevention of OM during cancer treatment. The study found a reduction in SOD activity in the control group, which was associated with higher OM incidence. In contrast, patients treated with PBMT did not experience the same reduction, suggesting that PBMT can modulate the expression of this enzyme.

The modulation of SOD activity by PBMT has been previously reported, with studies showing reduction in oxidative stress-induced damage and increased SOD activity following low-level laser irradiation in various conditions^39^^–^ 41. This highlights PBMT’s potential role in counteracting oxidative stress, thereby reducing the severity of OM and enhancing tissue recovery in cancer patients.

Antunes et al.^23^ conducted a pilot RCT to investigate keratinocyte differentiation in patients undergoing laser therapy, utilizing cDNA microarray analysis. The authors observed overexpression of the SPRR2B and SPRR2F genes in the laser-treated group. The SPRR gene family plays a well-recognized role in maintaining keratinocyte homeostasis. Studies indicate that these proteins facilitate cell migration and contribute to wound healing^42^. Previous evidence has demonstrated the effect of PBMT on keratinocytes, both *in vitro* and *in vivo*, suggesting that PBMT influences keratinocyte differentiation and accelerates tissue healing^43^^,^ 44.

The elucidation of the mechanisms of action of PBMT in the management of OM is of clinical importance to improve the treatment of patients. The action of the PBMT, on a specific target, allows us to understand the effectiveness of the laser in the different stages of OM. In addition, it can provide information for the association of PBMT with other therapies, with the use of drugs directed to signaling pathways that phototherapy does not act.

The main limitation of this review was the heterogeneity of the included studies. Differences in laser application parameters, treatment protocols, and methods of analytical sample collection were observed, which may have affected the consistency and comparability of the results. This variability makes it challenging to elucidate the underlying mechanisms and highlights the need to address this methodological diversity in future research. Additionally, the literature’s need for more RCTs represents another significant gap.

## Conclusion

Our study demonstrated that OM is a dynamic process influenced by various factors, with a complex pathophysiology involving multiple mediators. The findings suggested that the mechanism of action of PBMT in OM is related to the modulation of the inflammatory response, balancing the generation of ROS, and/or the expression of factors associated with healing and tissue repair. However, the data available in the literature have limitations, and the number of controlled clinical trials needs to be improved. Thus, further studies are required to elucidate better the mechanisms involved in the effects of PBMT.

## Data Availability

Data sharing is not applicable.
